# Influence of light-curing distance on degree of conversion and cytotoxicity of etch-and-rinse and self-etch adhesives

**DOI:** 10.1186/s12903-016-0239-3

**Published:** 2016-07-07

**Authors:** Florian J. Wegehaupt, Nancy Lunghi, Georgios N. Belibasakis, Thomas Attin

**Affiliations:** Clinic for Preventive Dentistry, Periodontology and Cariology, University of Zurich, Plattenstrasse 11, 8032 Zürich, Switzerland; Section of Oral Microbiology and Immunology, Institute of Oral Biology, University of Zürich, Plattenstrasse 11, 8032 Zürich, Switzerland

**Keywords:** Cytotoxicity, Degree of conversion, Dental adhesives, Light-curing

## Abstract

**Background:**

The degree of conversion (DC) of resin based materials depends, beside other factors, on the light-intensity applied during light curing. A lower DC might be correlated with an increased cytotoxicity of the respective materials.

Therefore, aim of the present study was to investigate the influence of the distance between light-curing tip and adhesives on their cytotoxicity and degree of conversion (DC).

**Methods:**

For the cytotoxicity assay, a total of 98 bovine dentine samples were prepared, distributed to seven groups (G1-G7; *n* = 14) and treated as follows: G1: untreated; G2-G4: OptiBond FL; G5-G7: OptiBond All-In-One. Adhesives were light-cured (1200 mW/cm^2^) at 1 mm (G2;G5), 4 mm (G3;G6) or 7 mm (G4;G7) distance. Samples were stored in culture media for 24 h and extracts were added to cell cultures (dental pulp cells and gingival fibroblasts) for a further 24 h. Finally, released lactate dehydrogenase activity (LDH) was photometrically determined, as measure for the cytotoxic effects of the extracts. The cytotoxicity assay was performed three times.

Additionally, the DC of the adhesives was determined by FTIR spectroscopy. DC measurements were performed five times.

**Results:**

For both cell types, no significant difference of LDH release was observed between untreated control group (G1) and treated groups G2-G7 (*p* > 0.05, respectively), between the groups treated with same adhesive and light-cured at different distance (*p* > 0.05, respectively), as well as between groups treated with different adhesives and light-cured at the same distance (*p* > 0.05, respectively). Within the respective adhesive, no significant difference in the DC was observed when light-cured at different distance (*p* > 0.05, respectively), while OptiBond FL showed significantly higher DCs compared to OptiBond All-In-One when light-cured at same distances (*p* < 0.05, respectively).

**Conclusions:**

The distance between light-curing tip and adhesive surface does not significantly influence either the cytotoxicity or the DC of the tested adhesives.

**Electronic supplementary material:**

The online version of this article (doi:10.1186/s12903-016-0239-3) contains supplementary material, which is available to authorized users.

## Background

The increased demand for anatomical and functional but also esthetical and minimal invasive tooth restorations resulted in a rapid further development of the adhesive dentistry [1]. This growth in importance and the usually needed close contact of dental adhesives with vital dental and oral tissues make biocompatibility one of the most important requirements for these materials [2].

Several in-vitro [3–6] and in-vivo [7–11] studies reported cytotoxic effects of dental adhesives [12] and liners [13]. The release of resin monomers being present in the adhesive systems has been implicated as possible cause for these adverse phenomena [7, 10]. Their cytotoxicity, defined as capacity to damage tissue cells [6], has been observed in various in-vitro-studies [14–16].

One of the factors that contribute to monomer release – and therefore indirectly to adhesive cytotoxicity – is the degree of conversion (DC) [14, 17–21], i.e. the extent to which carbon double bonds (C = C) of resin monomers are converted into carbon single bonds (C–C) to form polymers during the polymerization reaction of dental adhesives. This transformation is never completely achieved [17–22] and in case of light-cured polymerization is influenced by various parameters, such as light source, intensity, emission spectrum and exposure time, viscosity and thickness of adhesive layer, temperature, air inhibition, presence of solvents, concentrations, types and mixtures of photoinitiators, co-initiators, stabilizers and inhibitors, as well as types and proportions of monomers and fillers [17, 22–27]. The total amount of energy per unit area, the so-called energy density, which is the product of the light-intensity emitted by the light-curing unit and the exposure time, [28] is of particular importance for DC of light-cured adhesives. Generally, the higher the light-intensity and/or longer the exposure time, the higher the energy density applied to the resin-based material and the higher the DC will be [28].

Several studies revealed a correlation between the effective light-intensity available for the light-curing of resin monomers and the distance between the light-curing tip and the material surface, according to which the light-intensity may decrease proportional to the square of the distance, i.e. doubling the distance between the light-curing tip and the material surface results in a reduction of the light-intensity to one quarter of the original light-intensity [29–31].

Taking in consideration these finding, it seems to be conceivable that an increased distance between the light-curing tip and the adhesive surface, which decreases the light-intensity and consequently the energy density, should decrease the DC and finally result in an increased cytotoxicity of the dental adhesives.

Therefore, the aim of the present study was to investigate if the distance between light-curing tip and an etch-and-rinse and a self-etch adhesive has an influence on the cytotoxicity and DC of the adhesives.

The null-hypotheses of this study were that distance between light-curing tip and adhesive surface have no influence on either the cytotoxicity (I) and the DC (II) of etch-and-rinse and self-etch adhesives.

## Methods

The present study consists of two parts: the first includes a cytotoxicity assay to determine the biocompatibility of the different cured adhesives, whereas the second involves a spectroscopic measurement of degree of conversion of the so-cured adhesives.

### Cytotoxicity assay

For the cytotoxicity assay, three independent experiments were performed (including sample preparation, adhesive application and light-curing procedure, preparation of extracts and measurement of extracellularly released lactate dehydrogenase (LDH)).

### Sample preparation

A total of 98 dentine discs were prepared from freshly extracted bovine incisors. The bovine teeth were collected as anonymous by-products of regular slaughtering of the cattle. Slaughtering was performed to provide the cattle as foodstuff for human consumption. Therefore, no ethic approval was needed [32]. After removing the organic tissue and cleaning the teeth, dentine cores (5 mm in diameter) were extracted with a trephine drill from the distal and mesial surface of each root. These drilling cores were ground with a water-cooled abrasive paper disc (2500 grit; Water Proof Silicon Carbide Paper, Streuers, Erkrat, Germany) from the cementum side and milled from the pulp side to a thickness of 1 mm under constant water cooling. After preparation, the dentine discs were stored in water and gamma sterilized (12 kGy, 4 h, Paul Scherrer Institut, Villigen, Switzerland). The 98 dentine samples were randomly allocated to seven groups (0–6, *n* = 14).

### Adhesive application and light-curing procedure

The compositions of the products used in this study are given in Table 1.

**Table 1:** Composition of the used surface sealants (manufacturer ´s information)

Adhesive	Composition
OptiBond™ FL Primer	- Uncured methacrylate ester monomers: 2-hydroxyethyl methacrylate (HEMA), glycerol phosphate dimethacrylate (GPDMA) - 2-[2-(methacryloyloxy)ethoxycarbonyl]benzoic acid - Solvents: ethanol - Uncured methacrylate ester monomers: bisphenol A diglycidyl methacrylate (Bis-GMA), 2-hydroxyethyl methacrylate (HEMA), glycerol dimethacrylate (GDMA) - Triethylene glycol gimethacrylate (TEGDMA) - Inert mineral fillers: fumed silicon dioxide, barium aluminiumborosilicate, disodium hexafluorosilicate - Ytterbium trifluoride - Photoinitiator: camphorquinone Coupling factor A174
OptiBond™ FL Adhesive	
OptiBond®	- Uncured methacrylate ester monomers: glycerol phosphate dimethacrylate (GPDMA), glycerol dimethacrylate (GDMA), 2-hydroxyethyl methacrylate (HEMA), bisphenol A diglycidyl methacrylate (Bis-GMA) - Inert mineral fillers: nanosilicate, disodium hexafluorosilicate - Ytterbium fluoride - Photoinitiator: camphorquinone - Solvents: water, acetone and ethanol
All-In-One	

The dentine discs of group 0 were left untreated and served as control. The dentine discs of groups 1–3 were treated with OptiBond™ FL (Kerr Corporation, Orange, United States), an etch-and-rinse adhesive. The dentine was etched with phosphoric acid (37 %) for 15 s, then rinsed with water for 15 s. After rinsing, the dentine was carefully dried using an air syringe for 3 s and the primer was applied with light brushing motion for 15 s. Thereafter, the primer was air-dried for 5 s and the adhesive was applied with a light brushing motion for 15 s. Light curing was performed for 20 s after thinning the adhesive using an air syringe for 3 s. (manufactures’ instructions). The dentine discs of groups 4–6 where treated with OptiBond® All-In-One (Kerr Corporation, Orange, United States), a self-etch adhesive. A generous amount of OptiBond All-In-One adhesive was applied to the dentin with brushing motion for 20 s. Thereafter, the adhesive was applied for a second time, again with brushing motion, for 20 s. Finally, the adhesive was dried with gentle air first and then medium air for at least 5 s and than light-cured for 10 s (manufactures’ instructions).

The respective adhesives were light-cured with an intensity of 1200 mW/cm^2^ (Bluephase® G2, Ivoclar Vivadent AG, Schaan, Liechtenstein). In order to maintain an exact distance between light-curing tip and sample surface and at the same time to eliminate external irradiation sources, dark custom-made spacers were used. These spacers ensured a distance of 1 mm (groups 1 and 4), 4 mm (groups 2 and 5) or 7 mm (groups 3 and 6) between light-curing tip and the surface of the adhesives. The light-curing unit was checked for consistency prior and after curing using a radiometer (Optilux Radiometer, SDS Kerr; Orange, CA, USA). After light-curing the oxygen-inhibited layer was removed with a foam pellet.

### Preparation of extracts

The preparation of the extracts, cell cultures and measurement of extracellularly released LDH has been described in detail previously by Wegehaupt et al. [32].

In brief, the samples of each group were transferred into one well of a 12 well cell culture plate (SPL Life Sciences Inc., Gyeonggi-do, South Korea), taking care that the discs were placed with the treated side up in the well. Afterwards, dentine discs were covered with 3 ml cell culture medium (DMEM/F12 medium supplemented with 1 % penicillin/streptomycin, 1 % L-glutamine, 50 ng/ml fungizone and 10 % heat-inactivated foetal bovine serum; all from Sigma-Aldrich, Buchs, Switzerland) and incubated in the dark for 24 h at 37 °C and 5 % CO_2_, as performed in previous studies [32, 33]. This procedure results in a preparation of dentine disc extracts at a ratio of 91.6 mm^2^ sample surface per millilitre cell culture medium following the recommendations of ISO 10993 [33].

### Cell cultures

Human dental pulp cells from permanent teeth and gingival fibroblasts were obtained according to previously described procedures and ethical requirements [34, 35]. The gingival fibroblasts were provided by Dr. Anders Johansson, Institute of Odontology, Umeå University, Sweden (Human Studies Ethical Committee of Umeå University, Sweden - §68/03, dnr 03–029). The collection of dental pulp cells abides by guidelines of the Ethical Committee of the Canton of Zürich, Switzerland, for collection of material for research purposes obtained from discarded and irreversibly anonymized specimens of human origin [32]. For the present study, the cells were cultured in a cell culture medium (DMEM/F12 medium supplemented with 1 % penicillin/streptomycin, 1 % L-glutamine, 50 ng/ml fungizone and 10 % heat-inactivated foetal bovine serum) and the cell cultures of the seventh passage for the dental pulp cells and of the twelfth passage for the gingival fibroblasts were used. On a 96-well plate (TPP® tissue culture plate, Sigma-Aldrich, Buchs, Switzerland) four replicate cultures of each cell type were seeded for each one of the seven extract groups (0–6). Dental pulp cells were seeded at a density of 2 × 10^5^ cells per well, while gingival fibroblasts at a density of 1.2 × 10^5^ cells per well. After the seeding procedure, the cells were incubated in the dark for 24 h at 37 °C and 5 % CO_2_ to allow for cell attachment onto the bottom of the well, reaching 100 % confluence. Thereafter, 200 μl per well of the extracts were added to the cell cultures and incubated for 24 h.

### Measurement of extracellularly released LDH

The potential cytotoxic effects of the different treatment groups on dental pulp cells and gingival fibroblast cultures were evaluated by measurement of the extracellularly released cytosolic lactate dehydrogenase (LDH), using the CytoTox96® Non-Radioactive Cytotoxicity Assay (Promega Dübendorf, Switzerland). After the 24 h exposure of the cell cultures to the material extracts, the cell culture supernatants were collected, while the adherent cells were lysed by three repeated freeze-thaw cycles in each 200 μl of cell culture medium. Both the cell supernatants and lysates were centrifuged (Beckman GS-6 Series Centrifuge, Beckman Coulter, Brea, United States) at 1000 rpm for 5 min. Both, the cell supernatants and lysates were diluted in the cell culture medium at 1 : 4 and 1 : 10 ratios, respectively. The obtained solutions were transferred into wells of a 96-well plate (Nunc-Immuno™ MicroWell™, Sigma-Aldrich, Buchs, Switzerland), followed by addition of reaction solution and placed in the dark for 30 min at room temperature. The absorbance was measured at 490 nm in a microplate reader (Epoch microplate reader, Biotek, Lucerne, Switzerland), subtracting the corresponding background values from all samples.

The cytotoxicity results are expressed as percentage of extracellularly released LDH activity, calculated against total (intracellular and extracellular) LDH activity. This percentage corresponds to the relative amount of dead cells among the total cell population in the culture [32].

### Spectroscopic measurement of degree of conversion

The degree of conversion (DC) was evaluated using a Fourier transform infrared (FTIR) spectrophotometer (Agilent Cary 630 FTIR, Agilent Techno-logies Inc., Santa Clara, United States), composed of a horizontal zinc selenide (ZnSe) crystal with a resolution of 4 cm^−1^, in the attenuated total reflectance (ATR) sampling mode. One drop of the respective adhesives was applied on the surface of the ZnSe crystal and measured as un-polymerised sample. Afterwards the adhesive on the ZnSe crystal was light-cured (1200 mW/cm^2^) for 20 s (OptiBond™ FL; groups 1–3) or 10 s (OptiBond® All-In-One; groups 4–6) at a distance of 1 mm (groups 1 and 4), 4 mm (groups 2 and 5) or 7 mm (groups 3 and 6) and re-measured as polymerised sample. Again, the light-curing durations were chosen following the manufactures’ recommendations. In order to maintain the exact light-curing distance between light-curing tip and sample surface and at the same time to eliminate external irradiation sources, dark custom-made spacers were used. For each combination (adhesive and distance between adhesive and light-curing tip), five measurements were performed (*n* = 5). As in some other studies [36–38], the DC for each sample was determined using a baseline technique [39], considering the absorbance intensity of aliphatic C = C stretching vibration (peak height) at 1635 cm^−1^ and using, as internal standard, the symmetric aromatic ring stretching vibration at 1608 cm^−1^, from polymerised and unpolymerised samples.

DC (%) for each sample was calculated using the following formula [38]:

$$ DC\ \left(\%\right)=\left[1-\frac{\left[\mathrm{aliphatic}\ \left(1635\ {\mathrm{cm}}^{-1}\right)/\mathrm{aromatic}\ \left(1608\ {\mathrm{cm}}^{-1}\right)\right]\ \mathrm{polymerised}\ }{\left[ aliphatic\ \left(1635\ {\mathrm{cm}}^{-1}\right)/\mathrm{aromatic}\ \left(1608\ {\mathrm{cm}}^{-1}\right)\right]\ \mathrm{unpolymerised}}\right] \times 100 $$

Where $\left[\frac{\mathrm{aliphatic}\left(1635\ {\mathrm{cm}}^{-1}\right)/\mathrm{aromatic}{\left(1608\ {\mathrm{cm}}^{-1}\right)}_{\mathrm{polymerised}}}{\mathrm{aliphatic}\left(1635\ {\mathrm{cm}}^{-1}\right)/\mathrm{aromatic}{\left(1608\ {\mathrm{cm}}^{-1}\right)}_{\mathrm{unpolymerised}}}\right]$ represents the residual double bonds.

### Statistical analysis

For each of the three independent experiments of the cytotoxicity assay, the mean percentage of released LDH of the four biological replicates per group was calculated. For the statistical analysis, the mean percentage of all three experiments was thereafter calculated per each one of the experimental groups.

The data were encoded into a Microsoft Excel (Microsoft Corp., Redmond, United States) file.

The statistical analysis was then performed using the software program IBM® SPSS® Statistics Version 22 (International Business Machines Corp., Armonk, United States).

The assumption of normal distribution of errors was checked, using Kolmogorov-Smirnov and Shapiro-Wilk tests.

Statistical analysis was performed by 2-way ANOVA with the factors distance (between light-curing tip and adhesive surface) and adhesive separately for dental pulp cells and gingival fibroblasts followed by Scheffe’s post hoc tests. Also for the degree of conversion, the statistical analysis was performed by 2-way ANOVA with the factors distance (between light-curing tip and adhesive surface) and adhesive followed by Scheffe’s post hoc tests.

Additionally, multiple linear regressions and a covariance analysis were applied in order to investigate linear dependence of the outcomes (extracellularly released LDH activity from dental pulp cells and gingival fibroblasts and DC), with respect to the predictors (distance, adhesive and interaction of distance and adhesive (distance*adhesive)).

Level of significance was set at *p* < 0.05.

## Results

### Extracellularly released LDH activity from dental pulp cells

**Fig. 1:**
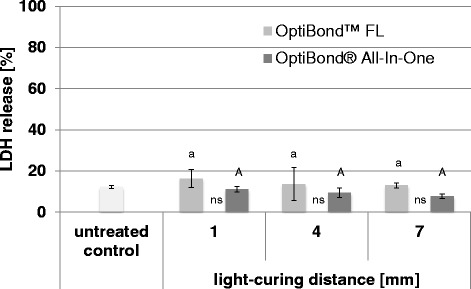
LDH release from dental pulp cells. Percentage (mean ± SD) of extracellularly released LDH activity from dental pulp cells for different adhesives (OptiBond FL and OptiBond All-In-One) and different light-curing distances (1 mm, 4 mm and 7 mm). Values of the test groups were not significantly different to the untreated control group. Comparisons within the same adhesives between the different light-curing distances that are not significantly different are marked with same letters (*lower case letters* and *capital letters* for OptiBond FL and OptiBond All-In-One, respectively). Comparisons within the same light-curing distance between the different adhesives that are not significantly different, are marked with ns

The percentage of extracellularly released LDH from pulp cells for the different groups treated with different adhesives and different light-curing distances are presented in Fig. 1.

The 2-way ANOVA showed a significant influence of the factor adhesive (*p* = 0.021) but for the factor distance no significant influence could be observed (*p* = 0.364).

All test groups showed no significantly increased cytotoxicity compared with the control group (*p* > 0.05, respectively).

A 2-way ANOVA showed a significant influence of the type of adhesive on the cytotoxicity. Within the respective light-curing distance, OptiBond™ FL showed a higher cytotoxicity than OptiBond® All-In-One, however these differences were not statistically significant (1 mm distance: *p* = 0.801; 4 mm distance: *p* = 0.900 and 7 mm distance: *p* = 0.779).

### Extracellularly released LDH activity from gingival fibroblasts

**Fig. 2:**
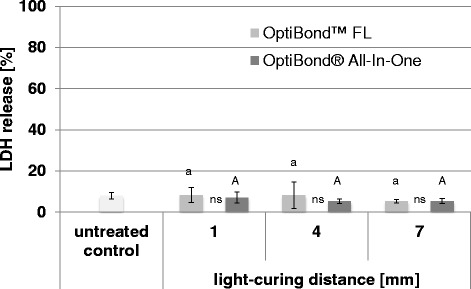
LDH release from gingival fibroblast. Percentage (mean ± SD) of extracellularly released LDH activity from gingival fibroblasts for different adhesives (OptiBond FL and OptiBond All-In-One) and different light-curing distances (1 mm, 4 mm and 7 mm). Values of the test groups were not significantly different to the untreated control group. Comparisons within the same adhesives between the different light-curing distances that are not significantly different are marked with same letters (*lower case letters* and *capital letters* for OptiBond FL and OptiBond All-In-One, respectively). Comparisons within the same light-curing distance between the different adhesives that are not significantly different, are marked with ns

The percentage of extracellularly released LDH from gingival fibroblasts for the different groups treated with different adhesives and different light-curing distances are presented in Fig. 2.

The 2-way ANOVA showed no significant influence of the two factors adhesive (*p* = 0.395) and distance (*p* = 0.513).

All test groups showed no significant higher cytotoxicity compared with the control group (*p* > 0.05, respectively).

### Degree of conversion

**Fig. 3:**
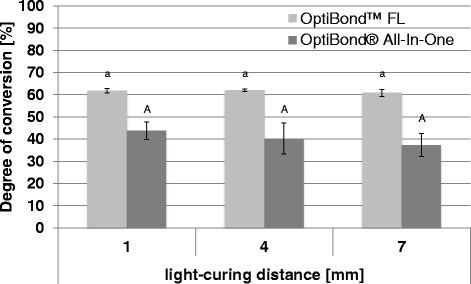
Degree of conversion. Degree of conversion [%] (mean ± SD) of the different adhesives (OptiBond FL and OptiBond All-In-One) cured at different light-curing distances (1 mm, 4 mm and 7 mm). Within the same adhesive, values for different light-curing distances, that are not significantly different, are marked with same letters (*lower case letters* and *capital letters* for OptiBond FL and OptiBond All-In-One, respectively). Comparisons within the same light-curing distance between the different adhesives that are not significantly different, are marked with ns

The degrees of conversion of the respective adhesives light-cured at different distances are presented in Fig. 3.

For the degree of conversion, the 2-way ANOVA showed a significant influence of the factor adhesive (*p* = 0.000) but for the factor distance no significant influence could be observed (*p* = 0.135). Within the respective distances, OptiBond™ FL showed a statistically significantly higher degree of conversion than OptiBond® All-In-One (*p* < 0.05, respectively).

### Multiple linear regressions and covariance analysis

The results of the multiple linear regressions (Covariance Analysis) are presented in Table 2.

**Table 2:** Results of the multiple linear regression and covariance analysis for the LDH release from dental pulp cells and gingival fibroblast and DC

	Pulp cells				Gingival fibroblast				DC			
Parameter	β	Std. error	*p*-value	95 % CI	β	Std. error	*p*-value	95 % CI	β	Std. error	*p*-value	95 % CI
Intercept	16.47	2.34	0.000	11.45	9.19	1.99	0.000	4.91	62.17	1.90	0.000	58.25
				21.48				13.47				66.08
Distance	−0.54	0.50	0.298	−1.61	−0.482	0.426	0.277	−1.39	−0.16	0.41	0.703	−0.99
				0.53				0.43				0.68
Adhesive	−4.82	3.31	0.168	−11.91	−2.22	2.82	0.446	−8.27	−17.41	2.69	0.000	−22.94
				2.28				3.84				−11.87
Distance×	−0.03	0.71	0.971	−1.54	0.21	0.60	0.732	−1.08	−0.93	0.57	0.118	−2.11
Adhesive				1.49				1.50				0.25

For the extracellularly released LDH activity from dental pulp cells and gingival fibroblasts no significant influence of the predictors distance, adhesive and interaction distance*adhesive was observed (*p* > 0.05, respectively).

For the DC, no significant influence of the predictors distance and interaction distance*adhesive was found (*p* = 0.703 and 0.118, respectively) while for the predictor adhesive a significant influence was observed (*p* = 0.000).

## Discussion

In the present study, the extracts of the adhesives were prepared by immersing in cell culture medium dentine discs covered with the different adhesives and light-cured at different distances.

The respective adhesives where applied on dentin discs as this reflects the clinical situation as close as possible. Other studies investigating the cytotoxicity of dental materials e.g. adhesives exposed cell culture media to the respective materials light-cured in vials [40, 41], wells [42], on glass slides [43, 44] or in moulds [45] or dissolved the uncured materials in cell culture medium [40, 46]. As dental adhesives are developed to react with dental hard tissues, it might be disadvantageous to test these materials not applied to dentine or enamel. It can be assumed that during the reaction of the adhesives with dental hard tissues there is a change in the chemical composition or reactiveness of the tested materials. This change might have an influence on the later release of possible cytotoxic compounds from the materials [32]. Furthermore, when curing these materials in vials or wells (especially a well of 96-well microplates) it might be difficult to remove the oxygen inhibition layer, rich on uncured monomers and adhesive components, on top of these materials. From the oxygen inhibition layer, uncured monomers and other components can be easily diluted, resulting in an increased amount of these substances in the extracts prepared from the corresponding samples.

In contrast to other studies [47–49], no intra-pulpal pressure was simulated during the preparation of the extracts. When an intra-pulpal pressure is simulated (dentine barrier test setup), this might decrease the contact of pulp cells with the adhesives applied on the dentine due to the outward directed flow of dentine fluid or cell culture media. Therefore, we assume that the respective values gained in the present study for the cytotoxicity on dental pulp cells might be over-estimated. In contrast, if the adhesives would have been tested in a dentine barrier test setup, there might have been an under-estimation of the cytotoxic effect on gingival fibroblasts. Under clinical conditions these cells might be in direct contact to the adhesives and not been protected by the dentine layer and the outward flow of the dentin fluid. Taking into account these considerations, an over-estimation of the cytotoxicity on dental pulp cells seems to be more acceptable than an under-estimation of the cytotoxic effect on gingival fibroblasts. Limitation of the present study might be, that up to now no information is available concerning the threshold concentrations of monomers that are able to trigger unwanted reaction during the long term clinical service of resin based material. Furthermore, the use of costume-made spacers deviates from the clinical situation, as they might focus the energy delivered and lead to false reporting versus a clinical scenario where this dark spacer is not used and where the light might be scattered by the surrounding dental hard tissues. However, we assume that the use of these spacers is favourable as they eliminate the chance of irradiation by indirect surrounding light.

Human dental pulp cells and gingiva fibroblasts were used for the here performed biocompatibility testing. Under clinical conditions these cell types are the ones mainly affected by the cytotoxicity of dental adhesives. Numerous other studies have used dental pulp cells [8, 41, 47, 48] and gingival fibroblasts [6, 42, 50, 51] while testing the cytotoxicity of dental adhesives.

Both null-hypotheses, that distance between light-curing tip and adhesive surface have no influence on either the cytotoxicity and the DC of etch-and-rinse and self-etch adhesives, have to be accepted. The results of this study in fact suggested that the distance between the light-curing tip and the adhesive surface does not significantly influence either the cytotoxicity or the DC of the here used etch-and-rinse and self-etch adhesives. These results correlate well to each other as the DC is in fact one of the factors that contribute to monomer release and also indirectly to adhesive cytotoxicity [14, 17–21]. Therefore, the not significantly influenced DC by means of different distance between light-curing tip and adhesive surface may in part explain the not significantly influenced cytotoxicity.

It is difficult to compare the findings of the present study with the literature, as up to date no study has examined the association between the light-curing distance and the cytotoxicity and the DC, within the same study, of dental adhesives or other similar resin-based materials.

However, a number of studies investigated the influence of light-curing distance on other parameters correlating with the polymerization effectiveness, such as light-intensity [29, 52], depth of cure [31] and surface hardness [30, 53] of light-cured composites and found that an increased distance between the light-curing tip and the composite surface may decrease the light-intensity, the depth of cure and the surface hardness – and consequently the polymerization effectiveness. These findings contrast the ones of the present study, in which the light curing-distance did not significantly influence the DC of the tested dental adhesives. A possible explanation of these contrary findings maybe attributed to differences in filler proportion, thickness and viscosity between composites and adhesives [6, 54–56]. The less filled, thinner and low viscous adhesives could may enable a similar light-polymerisation independently from the light-curing distances used in this study (1, 4 or 7 mm).

The present results are corroborated by a study evaluating the influence of light-curing distance (0, 3 or 6 mm) on bond strength and nanoleakage of self-etching adhesives [57]. In this study the light-curing distance does not influence either the bond strength or the nanoleakage on dentine substrates, which may give an allusion of proper DC values. Previous study observed that DC influences the nanoleakage [58] occurring within dental adhesives after polymerisation. The not significant influence of the distance of light-curing tip on DC as found in the present study, could therefore explain the unchanged nanoleakage on dentine surface, after polymerisation with different light-curing distances, as observed by Pimenta de Araujo et al. [57]. Additionally, it is possible that the not significantly influenced DC between the groups with different distances between light-curing tip and adhesive surface, have to be attributed to the here used light-curing unit. It might be assumed that the here used light-curing unit applies, even at a distance of 7 mm between light-curing tip and adhesive surface, a sufficient amount of light intensity to adequately activate the adhesive system resulting in a fair DC. Due to this assumption, the findings of the present study might be significantly different if a different light-curing unit with a lower light-intensity would have been used.

The results of the present study indicate that neither of the tested adhesives showed a significantly increased cytotoxicity compared to the untreated control group, nor was there any differential response between dental pulp cells and gingival fibroblasts. These findings contrast with that of other in vitro studies, in which dental adhesives resulted in increased cytotoxic effects [3, 4, 8, 9, 59]. In part, this discrepancy may be explained through the fact that in the present study, in contrast to the above mentioned studies, the oxygen-inhibited layer was removed from the adhesive surface. As explained previously, failure of removal of the oxygen-inhibited layer may lead to an over-estimation of the adhesive cytotoxicity [22, 32].

Ulterior findings of this study regard the influence of the type of dental adhesive on cytotoxicity and DC. According to the results it might be assumed that the type of adhesive have an influence on the DC: at the same light-curing distance, the etch-and-rinse adhesive tested showed higher DC values than the self-etch-adhesive. The main cause for this difference may be attributed to the different composition of the two adhesives. This might be assumed due to the finding that no differences in the cytotoxicity of the different types of adhesives were observed, while there was a difference in the DC. Thus factors other than polymerization (DC) might have also influenced the cytotoxic properties of dental adhesives [60].

The findings of the present study are encouraging with regard to proper light-curing of adhesives also in deep cavities. Nevertheless, it is still recommended to place the light-curing tip as close as possible to the adhesive surface in order to reach optimum polymerization. However, this approach is often difficult to achieve in practice and it is not uncommon, e.g. in deep class II cavities, to have distances greater than 6 mm between the light-curing tip and the gingival wall of the proximal box. In extreme clinical situations, for example in a distal box of a second molar, the distance between light-curing tip and adhesives surface might be even greater than the here tested 7 mm. To evaluate the situation in such deep cavities, further studies are needed. In case that with such deep cavities a negative influence of the distance between light-curing tip and adhesives surface would be observed, it is conceivable to use dual cure adhesive systems to avoid an insufficient curing (unacceptable decreased DC) resulting in a possible increased cytotoxic effect of the adhesives.

## Conclusion

Within the limitations of the present study (chosen light-curing unit, adhesives and distances between light-curing tip and adhesives surface), it can be concluded that the distance between the light-curing tip and the adhesive surface does not significantly influence either the cytotoxicity or the DC of the here tested etch-and-rinse and self-etch adhesives. Furthermore, both tested adhesives show no significantly increased cytotoxicity compared to the untreated control group. Hence, the type of adhesive may significantly influence the DC, but not the cytotoxicity.

Further studies are needed to evaluate the effect of the distance between light-curing tip and adhesive surface for more extreme distances (e.g. exceeding 7 mm) and/or light-curing units with different light-intensities.

## Additional file

Additional file 1: Data for Statistics Lunghi. Raw data supporting the findings and conclusion of the present study. Raw data for the extracellularly released LDH activity from dental pulp cells and gingival fibroblasts as well as raw data for the DC of conversion in the different groups. (XLSX 41 kb)
